# Glutaredoxin 2 Protein (Grx2) as an Independent Prognostic Factor Associated with the Survival of Colon Adenocarcinoma Patients

**DOI:** 10.3390/ijms25021060

**Published:** 2024-01-15

**Authors:** Marlena Brzozowa-Zasada, Adam Piecuch, Karolina Bajdak-Rusinek, Karolina Gołąbek, Marek Michalski, Kamil Janelt, Natalia Matysiak

**Affiliations:** 1 Department of Histology and Cell Pathology in Zabrze, Faculty of Medical Sciences in Zabrze, Medical University of Silesia, 40-055 Katowice, Polandnmatysiak@sum.edu.pl (N.M.); 2 Department of Medical Genetics, Faculty of Medical Sciences in Katowice, Medical University of Silesia, 40-055 Katowice, Poland; 3 Department of Medical and Molecular Biology, Faculty of Medical Sciences in Zabrze, Medical University of Silesia, Jordana 19, 41-808 Zabrze, Poland; 4 Zabrze Silesian Nanomicroscopy Centre in Zabrze, Silesia LabMed-Research and Implementation Centre, Medical University of Silesia, 40-055 Katowice, Poland

**Keywords:** glutaredoxins, redox balance, colon adenocarcinoma, glutathione (GSH), prognostic factor, oxidative stress, S-gluathionylation, Grx2, ELISA

## Abstract

Glutaredoxin 2 (Grx2; Glrx2) is a glutathione-dependent oxidoreductase located in mitochondria, which is central to the regulation of glutathione homeostasis and mitochondrial redox, and plays a crucial role in highly metabolic tissues. In response to mitochondrial redox signals and oxidative stress, Grx2 can catalyze the oxidation and S-glutathionylation of membrane-bound thiol proteins in mitochondria. Therefore, it can have a significant impact on cancer development. To investigate this further, we performed an immunohistochemical analysis of Grx2 protein expression in colon adenocarcinoma samples collected from patients with primary colon adenocarcinoma (stage I and II) and patients with metastasis to regional lymph nodes (stage III). The results of our study revealed a significant relationship between the immunohistochemical expression of Grx2 and tumor histological grade, depth of invasion, regional lymph node involvement, angioinvasion, staging, and PCNA immunohistochemical expression. It was found that 87% of patients with stage I had high levels of Grx2 expression. In contrast, only 33% of patients with stage II and 1% of patients with stage III had high levels of Grx2 expression. Moreover, the multivariate analysis revealed that the immunohistochemical expression of Grx2 protein apart from the grade of tumor differentiation was an independent prognostic factors for the survival of patients with colon adenocarcinoma. Studies analyzing Grx2 levels in patients’ blood confirmed that the highest levels of serum Grx2 protein was also found in stage I patients, which was reflected in the survival curves. A higher level of Grx2 in the serum has been associated with a more favorable outcome. These results were supported by in vitro analysis conducted on colorectal cancer cell lines that corresponded to stages I, II, and III of colorectal cancer, using qRT-PCR and Western Blot.

## 1. Introduction

Colon adenocarcinoma (COAD) is a common type of colorectal cancer (CRC) that has become more prevalent in recent years. It is a complex malignancy with a high chance of recurrence and a poor prognosis. Unfortunately, there are currently limited effective therapeutic strategies available to treat recurrent and metastatic COAD. Developing a new prognostic tool to identify patients at a high risk of relapse who need extra attention and treatment is therefore crucial [1,2]. The time of COAD identification is linked to how the disease will develop, showing that it is very important to find it early. Screening tests carried out during early stages of the disease can lead to a very high 5-year survival rate of 90% for 40% of patients. However, 60% of cases in advanced stages (stage III–IV) go unnoticed, which leads to a poor prognosis and more deaths [3].

Because of their rapid proliferation and capacity to survive and adapt to various external conditions and cytotoxic therapy, cancer cells have great metabolic needs. Reactive oxygen species (ROS) are a major contributor to this. ROS are formed when electrons emitted from fuel oxidation affect the balance of reduced and oxidized electron acceptor pairs, for example, NADH/NAD+ and NADPH/NADP+. Electrons lost from fuel oxidation can bind with oxygen to generate ROS [4]. Cells have acquired several ways to guard against reactive oxygen species (ROS), which may damage DNA, proteins, and lipids. In cancer cells, which have a very high redox reaction rate, these protection systems are especially critical. Glutathione (GSH), which is present in every cell compartment at levels ranging from 1 to 10 mM, is involved in a number of these anti-ROS defense pathways. GSH is vital for the renewal of both enzymatic and non-enzymatic antioxidants. This involves the renewal of glutathione peroxidases (GPX), which helps oxidize lipid hydroperoxides and H_2_O_2_. It is also used by glutathione S-transferase (GST) enzymes for the detoxification of exogenous substances or oxidative stress-induced products [5]. The reversible glutathionylation/deglutathionylation reactions catalyzed by glutaredoxin (GRX) enzymes also directly use GSH [6]. This also helps in the non-enzymatic formation of alpha-tocopherol, which protects against the lipid peroxidation of cell membranes. GSH can also neutralize superoxide anion radicals directly [6]. Since GSH is necessary to enable all these processes, it is recycled via reactions that include those carried out by the enzyme glutathione reductase (GR), requiring NADPH as a coenzyme [7]. 

Increased GSH levels in many normal and neoplastic cells are linked to proliferative stimuli and are critical for progression through the cell cycle. An important role of GSH in DNA synthesis involves the maintenance of reduced glutaredoxin or thioredoxin, which is necessary for the action of ribonucleotide reductase, one of the rate-limiting DNA synthesis enzymes [8,9]. Furthermore, in liver cancer and metastatic melanoma cells, GSH status is correlated with growth, and it has also been demonstrated that a direct correlation between GSH levels associated with cellular proliferation and metastatic activity exists [10]. Additionally, maintaining mitochondrial GSH homeostasis could be a critical survival factor for metastatic cells in the period directly following intra-sinusoidal arrest and subsequent interaction with vascular endothelial cells. The disruption of mitochondrial GSH import may play a key function in sensitizing metastatic cancer cells to pro-oxidant agents that can activate cell death machinery. It has been shown that cancer cell lines with low levels of GSH are considerably more sensitive to the action of radiotherapy compared with the reference cells [11]. In certain types of cancer, the antioxidant function of glutathione may become unnecessary due to the thioredoxin antioxidant pathway fulfilling the antioxidant requirements. This makes GSH inhibition alone ineffective for cancer treatment. However, GSH plays an important role in chemotherapy resistance, which means that inhibiting GSH in combination with prodrug treatments can significantly improve the efficacy of chemotherapies [4]. 

Glutaredoxins (Grxs; Glrxs) are small proteins found in both prokaryotes and eukaryotes. They have a size of around 12 kD and act as oxidoreductases. Their primary function is to reduce disulfides and mixed disulfides that involve a protein’s thiol and glutathione [12,13]. Grxs can reduce disulfide bridges using a similar dithiol mechanism as thioredoxins (Trxs), but they can also use a monothiol mechanism that only requires one of the two active site cysteines [14]. According to recent studies, protein–GSH adducts and Grxs are crucial in balancing certain chemicals in the body. This balance is essential since any imbalances could lead to diseases, such as asthma and chronic obstructive pulmonary diseases [15], fatty liver disease [16], neurodegenerative diseases [17,18,19], cardiovascular disease [20], and cancer [21]. Glutaredoxin 2 (Grx2; Glrx2) is an enzyme that is of special interest in cancer research. It has been found that Grx2 can act as a backup for the reduction in Trx when cells are exposed to electrophiles that are strong inhibitors of thioredoxin reductase (TrxR). Grx2 is the enzyme previously found in mitochondria, the major cellular reservoir of ROS [22]. Grx2 can catalyze the oxidation and glutathionylation of mitochondrial thiol proteins, protecting complex I activity and inhibiting H_2_O_2_-induced apoptosis [23,24]. Furthermore, Grx2 can detoxify the mitochondrial mutant superoxide dismutase 1 (SOD1) by preventing its aggregation [25]. It has been observed that HeLa cells that overexpress Grx2 are more resistant to oxidative stress inducers. However, siRNA knockdown of GRX2 gene sensitizes HeLa cells to treatment [26]. These findings indicate that mitochondrial Grx2 is crucial for both cell survival and apoptosis [27]. All of the above suggests that the changes in *GRX2* expression may be important in the context of cancer development. Nevertheless, there is limited information available in the literature about the expression of *GRX2* in the development of cancer. Moreover, there are no data associated with the role of Grx2 protein gastrointestinal tract. As a result, we conducted a study to investigate the immunohistochemical expression of Grx2 protein in samples of colon adenocarcinoma taken from patients in stages I, II, and III. Our focus was on patients in the early stages, since most colorectal cancer patients are diagnosed at an advanced stage. To improve early diagnosis and treatment, it is essential to identify markers for early detection. The selection of our study group is therefore appropriate. We correlated the results of the immunohistochemical expression of Grx2 protein with patients’ clinical data and survival time to assess its potential as a biomarker. Furthermore, we aimed to confirm the localization of Grx2 protein in tumor tissue, which could be the foundation for future research related to the development of specialized cell therapy. Since cancer tissue is a highly diverse environment consisting of cancer cells and cells in the cancer microenvironment, we decided to conduct in vitro studies using colorectal cancer cell lines. This will enable us to confirm the expression of Grx2 protein solely in cancer cells without any exposure to signals from other parts of the tumor microenvironment, unlike in vivo tissue. To achieve this, we utilized q-RT-PCR and Western Blot techniques. The results of these studies will provide a basis for further molecular in vitro studies concerning *GRX2* gene expression. We are currently at a stage in our research where we need to check if the findings obtained from immunohistochemical techniques used on patients and samples of colon adenocarcinomas match the ones obtained from in vitro studies on the cell lines of stage I, II, and III patients. We also conducted an ELISA analysis of Grx2 serum levels in patients, and correlated these results with clinical data and patient survival.

## 2. Results

### 2.1. Patient Characteristics

In the study, we used the specimens obtained from 139 patients, with 69 being men and 70 being women. The average age of the participants was 64 years, with an age range of 54 to 77 years. Out of all the cases, 64 (46.04%) had cancers on the right side of the colon, while 75 (53.96%) had cancers on the left side. The histological grades of differentiation were classified into three categories: G1 with 26 cases (18.70%), G2 with 72 cases (51.80%), and G3 with 41 cases (29.50%). Regarding the depth of invasion (T parameter), 23 patients (16.55) were characterized as T1, 17 (12.23%) as T2, 78 (56.11%) as T3, and 21 (15.11%) as T4. Considering the stage of disease, 26 (18.17%) were described as those with stage I, 35 (25.18%) as those with stage II, and 78 (56.12%) as stage III (Table 1). 

**Figure 1:**
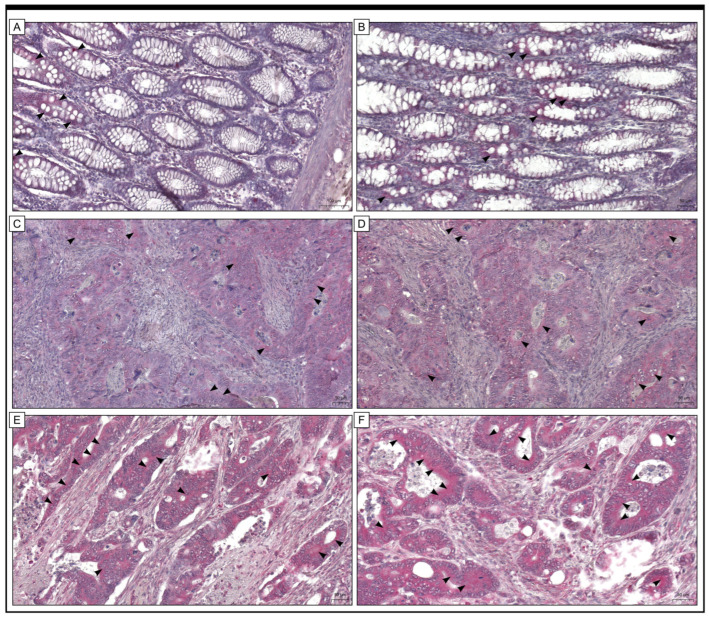
The immunohistochemical expression of Grx2 was detected in adjacent non-cancerous tissue margins (**A**,**B**) and colon adenocarcinoma tissue (**C**–**F**). The scale bar is 100 µm for (**A**) and 50 µm for (**B**–**F**).

Grx2 protein was observed in both cancer cells and stromal cells in the colon adenocarcinoma samples. A positive reaction was also detected in the cells of non-pathological colon tissue. Moreover, the expression of the Grx2 protein was found to be strong in most colon adenocarcinoma tissues, while it was low in cells of the adjacent non-pathological colon mucosa. Out of the group being studied, 70 samples of colon adenocarcinoma (which is 50.36%) showed high levels of Grx2 protein expression when examined immunohistochemically. In comparison, 69 samples (which is 49.64%) had low levels of immunoreactivity (Figure 1). 

### 2.2. Correlations between Patients’ Grx2 Tissue Immunohistochemical Expression (Dependent Variable) and Clinicopathological Parameters (Independent Variables)

The next step was to compare the results of the analysis of the immunohistochemical expression of Grx3 to the clinicopathological features which were treated as independent variables of patients and their survival in 60 months (5 years). It was found that Grx2 expression had a significant correlation with the histological grade of the tumor (*p* < 0.001, Chi^2^ test). The high levels of Grx2 protein expression were found in 24 samples (92.31%), 43 samples (59.72%), and 3 samples (7.32%) of G1, G2, and G3 tumors, respectively. It is worth noting that there was a significant association between Grx2 expression and angioinvasion (*p* < 0.001, Chi^2^ test). In patients with positive angioinvasion, 42 (39.25%) had high Grx2 immunohistochemical expression, while 65 (60.75%) had low immunoreactivity. On the other hand, among patients without angioinvasion, 28 (87.50%) had high Grx2 expression, while 4 (12.50%) had low Grx2 immunoreactivity. Furthermore, Grx2 protein expression was related to the depth of invasion (T) (*p* = 0.001, Chi^2^ test). Among T1 patients, a high level of immunohistochemical reaction was observed in 19 patients (82.61%), while 4 patients (17.39%) had a low level of expression. For T2 patients, 11 (64.71%) had a strong immunohistochemical reaction for Grx2, while 6 (35.29%) had a low level of expression. According to the study, the T3 group had 33 patients (42.31%) with high expression of Grx2, while the T4 group had 7 patients (33.33%) with high expression. In patients with stage I of the disease, 22 individuals (84.62%) showed high levels of Grx2 expression, while only 4 (15.38%) had low expression. For those with stage II of the disease, 11 patients (31.43%) had high Grx2 expression, while 24 (68.57%) had low expression. In patients with stage III of the disease, the expression levels varied, with 37 individuals (47.44%) having high expression and 41 (52.56%) having low expression (*p* < 0.001, Chi^2^ test) (Table 2).

When the immunohistochemical expressions of PCNA and Grx2 proteins were treated as dependent variables, a statistically significant relationship was found between these variables. In the study group, there were more cases with both high levels of PCNA and Grx2 (31.65%) of the total than subjects with concomitantly low levels of both PCNA and Grx2 (11.51%). There were also significantly more cases with both high levels of PCNA and low levels of Grx2 (38.13% of the total) than subjects with low levels of PCNA and high levels of Grx2 (18.71% of the total) (Table 3).

### 2.3. Prognostic Role of Grx2 Expression in Colon Adenocarcinoma

**Figure 2:**
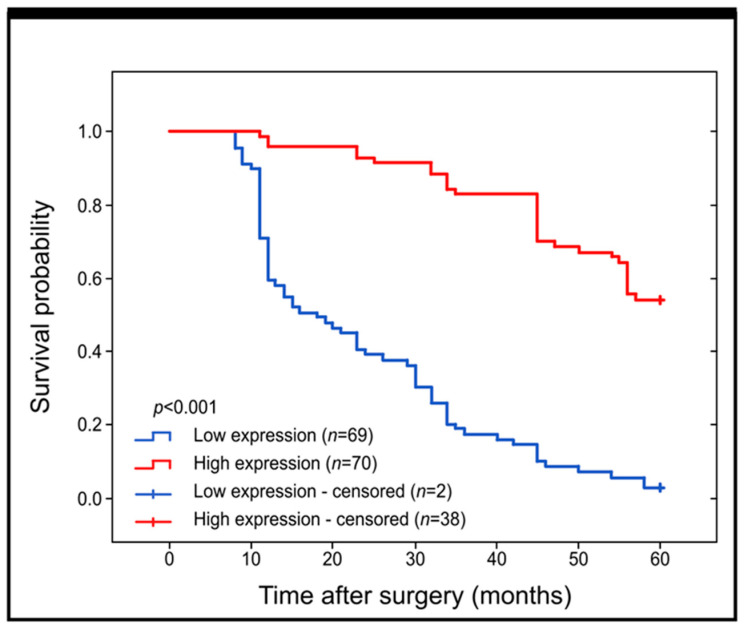
Kaplan–Meier curves comparing the survival probability for patients with high and low levels of Grx2 immunohistochemical expression.

Our study aimed to determine whether the expression of Grx2 has any impact on the survival of patients with colon adenocarcinoma. We used the Kaplan–Meier survival curves to analyze all samples. Our findings indicate that patients with a high expression of Grx2 had a significantly better prognosis than those with a low expression of this protein (log-rank, *p* < 0.001) (Figure 2). 

**Figure 3:**
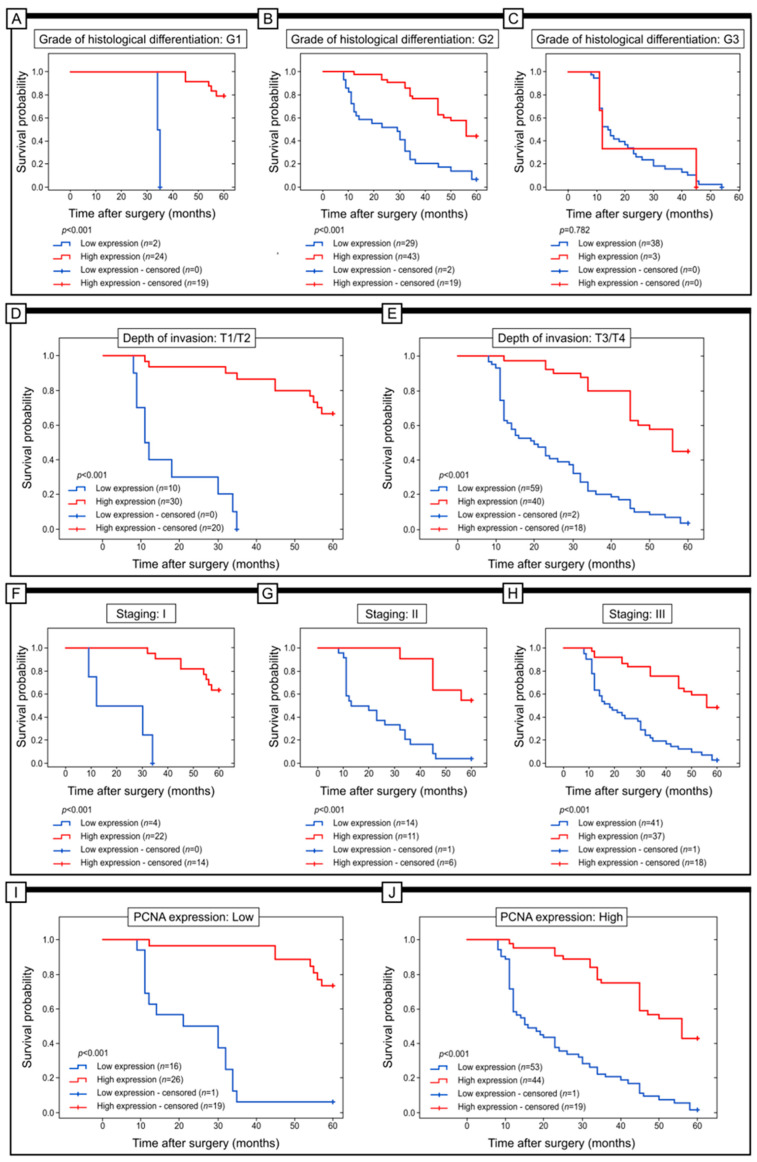
Kaplan–Meier curves were used to analyze the data on the expression of Grx2 protein expression in colon adenocarcinoma patients. The analysis was conducted through a log-rank test to compare patients with high versus low levels of Grx2 expression. The results were presented for patients with different grades of differentiation (G1, G2, and G3) (**A**–**C**), depth of invasion (T1/T2 and T3/T4) (**D**,**E**), and stages (I, II, and III) (**F**–**H**). Additionally, the analysis also compared patients with low and high expression of PCNA (**I**,**J**).

The study examined the relationship between the expression of Grx2 and the survival of patients in different subgroups. Factors such as histological differentiation, depth of invasion, staging, and PCNA immunohistochemical expression were taken into account. The results indicated that Grx2 expression did not significantly affect the survival of patients in the G3 group (log-rank test; *p* = 0.782). However, in patients categorized as G1 and G2, higher levels of Grx2 protein expression were associated with a better prognosis (log-rank test; *p* < 0.001). Comparable findings were observed in patients with T1/T2 and T3/T4 depth of invasion, as well as in those with I, II, and III stages of the disease. In these cases, higher levels of Grx2 expression were linked to better clinical outcomes (log-rank test; all *p* < 0.001) (Figure 3).

Our study examined various factors that may impact the survival of patients with colon adenocarcinoma. Through Cox regression analyses, we found that several factors, including Grx2 immunohistochemical expression, histological differentiation grade, invasion depth, angioinvasion, staging, and PCNA expression, were all significant prognostic factors. Multivariate analysis revealed that the grade of tumor differentiation and immunohistochemical expression of Grx2 were independent prognostic factors for the survival of patients with colon adenocarcinoma, as shown in Table 4 of our patient cohort data.

### 2.4. Immunofluorescence Staining

**Figure 4:**
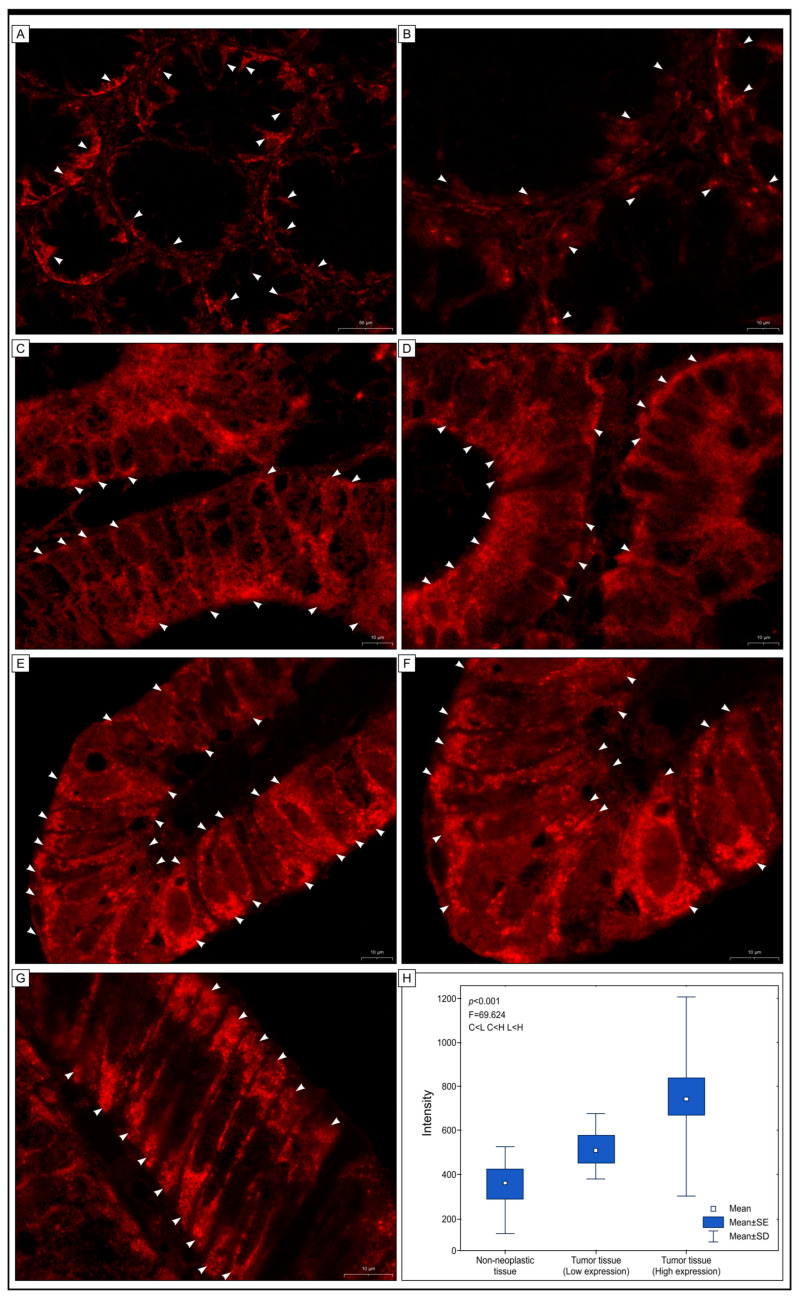
Immunofluorescence images were taken to evaluate the expression of Grx2 protein in colon adenocarcinoma (**C**–**G**) and adjacent non-carcinoma tissue (**A**,**B**). The images showed expression of the protein in the cytoplasm of colonocytes and cancer cells (**C**–**G**), represented by arrowheads. A red fluorescent signal was observed in cells located in the non-cancerous mucosa (**A**,**B**). In many cancer cells, the expression of Grx2 was observed in the cytoplasm of the apical regions of the cells, while in others, the fluorescence was strong and present throughout the cytoplasm of the cells or in the nuclei. Additionally, the results of the ANOVA test (**H**) showed differences in the intensity of the red signal, indicating the presence of Grx2 among the groups tested: C < H, L < H. Here, C represents colon tissue showing no pathological abnormalities, L represents adenocarcinoma samples with low expression of Grx2, and H represents adenocarcinoma tissue with a high expression of Grx2.

As per previous studies [28,29,30], our objective was to investigate the expression of Grx2 protein in colon adenocarcinomas via immunofluorescence staining. For this purpose, we selected 50 tissue sections that were stained with anti-Grx2 antibody and Dako Liquid Permanent Red chromogen (LPR). The slides included 10 control samples, 25 low-expression samples identified by immunohistochemical staining, and 25 high-expressing samples. While this method served as an add-on measure, the findings indicated that LPR chromogen application to anti-Grx2 antibody-stained tissue sections may be beneficial in the context of immunofluorescence studies. Zen 2 (blue edition) software was used to measure Grx2 expression levels in normal and cancerous tissues. Fluorescent signals were detected in both non-cancerous and cancerous colon cells, including in samples that showed no pathological changes. The signal was similar in both types of cells but at varying intensities. In some cancer cells, the signal was found in the apical cytoplasmic regions, while in others it was intense throughout the cytoplasm and nuclei (see Figure 4 for a visual representation).

### 2.5. The Localization of Grx2 Protein within Cell Compartments Using the Immunogold Labeling Method with TEM

**Figure 5:**
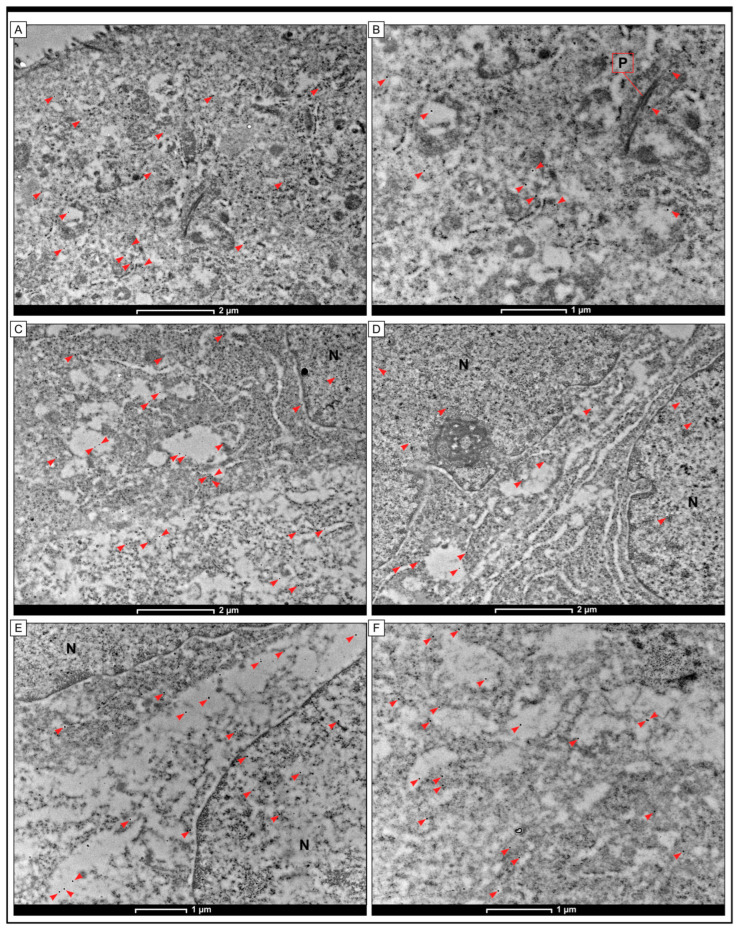
Detection of Grx2 protein via immunogold labeling at the level of transmission electron microscopy (TEM) in cells of colon adenocarcinoma tissue. The small electron-dense granules (red arrowheads) were found in adjacent non-tumoral cells (**A**,**B**) and cells of colon adenocarcinoma samples (**C**,**D**). In non-pathological samples, a limited amount of electron-dense aggregates were seen in the cytoplasm and the plasma membrane (P) close to adherent junctions. In cancer cells, the electron-dense aggregates associated with the localization of Grx2 were found in the cytoplasm within the mitochondria and cisterns of the endoplasmic reticulum. In a few cells, the aggregates were also found in the nucleus (N). The scale bar is 2 µm for (**A**,**C**,**D**) and 1 µm for (**B**,**E**,**F**).

The Grx2 protein has been detected in colon adenocarcinoma cells via immunogold labeling. The results indicated that black granules, which indicate the presence of Grx2 protein, were found in the cytoplasm and nucleus of cancer cells. Furthermore, electron-dense granules were observed in the mitochondria and cisterns of the rough endoplasmic reticulum. Scattered black granules were also seen in the cytoplasm of apical cells and the plasma membrane of human colon cells obtained from non-cancerous samples (Figure 5). 

### 2.6. GRX2 Gene Expression in Colorectal Cancer Cell Lines

**Figure 6:**
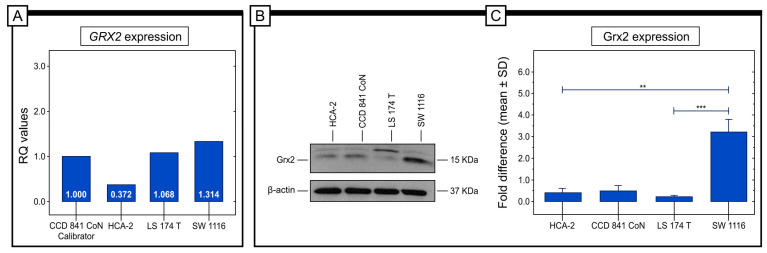
The purpose of this study was to examine the expression of the *GRX2* gene and Grx2 protein in different types of colorectal cancer cell lines. The relative quantification (RQ) gene expression level of *GRX2* was analyzed in various colorectal cancer cell lines (**A**). The protein expression levels of Grx2 were determined using the Western blot method (**B**), and the results showed that the highest level of Grx2 protein expression was in the SW 1116 cell line, while the lowest was in the LS 174 T cell line (**C**). The statistical significance of the *p*-values was represented as follows: ** *p*  <  0.01; *** *p*  <  0.001.

In order to investigate the relative gene expression (RQ) of the *GRX2* gene under in vitro conditions, various colorectal cancer cell lines were selected for testing, including SW 1116 (Duke A), LS 174T (Duke B), and HCA-2 (Duke C). The comparative threshold cycle (Ct) method 2-∆∆Ct was used, with the CCD 841 CoN line being used as the calibrator. Real-time PCR analysis was conducted, revealing that the highest level of *GRX2* gene expression was detected in SW 1116 (Duke A; Stage I) cells. Lower levels of *GRX2* gene expression were found in LS 174T (Duke B; Stage II) and HCA-2 (Duke C; Stage III) cells (Figure 6).

The protein expression levels in cancer cell lines were evaluated using the Western blot technique in vitro. The results indicated that Grx2 protein expression was the highest in the SW 1116 cell line, whereas LS 174T cells and HCA-2 cells exhibited low levels of expression. Figure 5 shows that significant differences in Grx2 protein expression were observed between SW 1116 and LS 174T, as well as between SW 1116 and HCA-2 cell lines (Figure 6).

### 2.7. The Preoperative Concentration of Grx2 in the Serum of Patients with Colon Adenocarcinoma

**Figure 7:**
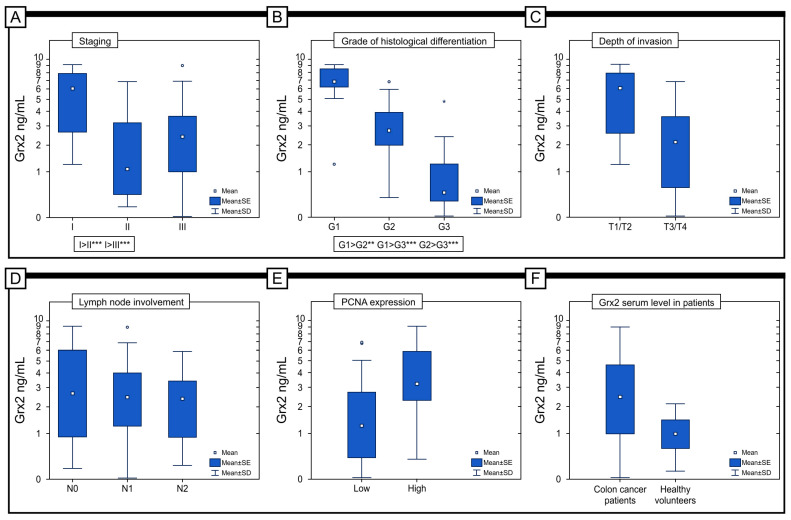
Comparison of serum Grx2 concentrations in colon adenocarcinoma patients according to the staging (**A**), grade of histological differentiation (**B**), depth of invasion (**C**), lymph node involvement (**D**), and immunohistochemical expression of PCNA protein (**E**). (**F**) Concentration of the serum level in colon adenocarcinoma patients and healthy volunteers. * *p* < 0.05, ** *p* < 0.01, *** *p* < 0.001.

The concentration of Grx2 in the serum of patients with colon adenocarcinoma (M = 3.06 ng/mL; Me = 2.49 ng/mL) was significantly higher than in the serum of healthy volunteers (M = 1.07 ng/mL; Me = 0.99 ng/mL) (*p* = 0.001) (Figure 7F). In addition, there was a statistically significant difference in the level of Grx2 in serum between patients with different stages of the disease, with the highest level of Grx2 detected in patients with stage I disease (M = 5.40 ng/mL; Me = 6.01 ng/mL) and the lowest level detected in patients with stage II disease (M = 2.02 ng/mL; Me = 1.07 ng/mL) (*p* < 0.001). In the case of histological differentiation, the highest level was found in patients in the G1 group (M = 6.83 ng/mL; Me = 6.84 ng/mL) and the lowest level was found in the G3 group (M = 0.96 ng/mL; Me = 0.46 ng/mL) (*p* < 0.001). Importantly, significantly higher levels were found in patients with T1/T2 (M = 5.36 versus M = 2.26 ng/mL) (*p* < 0.001) and N0 disease (M = 3.55 versus 2.99 versus 2.42 ng/mL) (*p* = 0.599), respectively, for the criteria of the depth of invasion (T) and lymph node involvement (N). In our study, we also tried to answer the question of whether the expression of Grx2 in tumor tissue correlates with the Grx2 content in the serum of patients. The study showed that a higher serum level of Grx2 characterizes patients with a high expression of Grx2 in the colon adenocarcinoma samples (M = 3.86 versus M = 1.94 ng/mL) (*p* < 0.001). 

**Figure 8:**
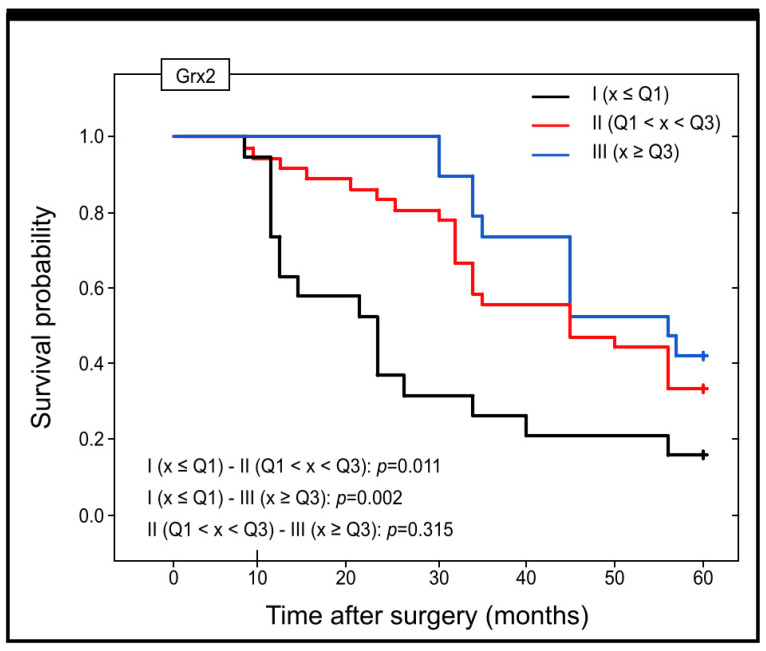
Kaplan–Meier curves showing the survival probability and its changes within the 60 months of the follow-up period for patients with different levels of Grx2 concentration in the serum of colon adenocarcinoma patients.

Taking into account the concentration of Grx2 in the serum of patients and its prognostic significance, it should be pointed out that there was a statistically significant difference in the estimated survival time between patients with low levels of Grx2 in blood serum (I group) and those with high levels of this protein (II group) (OS Me = 23 versus OS Me = 45) (*p* = 0.011). A significant difference was also detected between patients in groups I and III (OS Me = 23 versus OS Me = 56) (*p* = 0.02) (Figure 8). 

**Figure 9:**
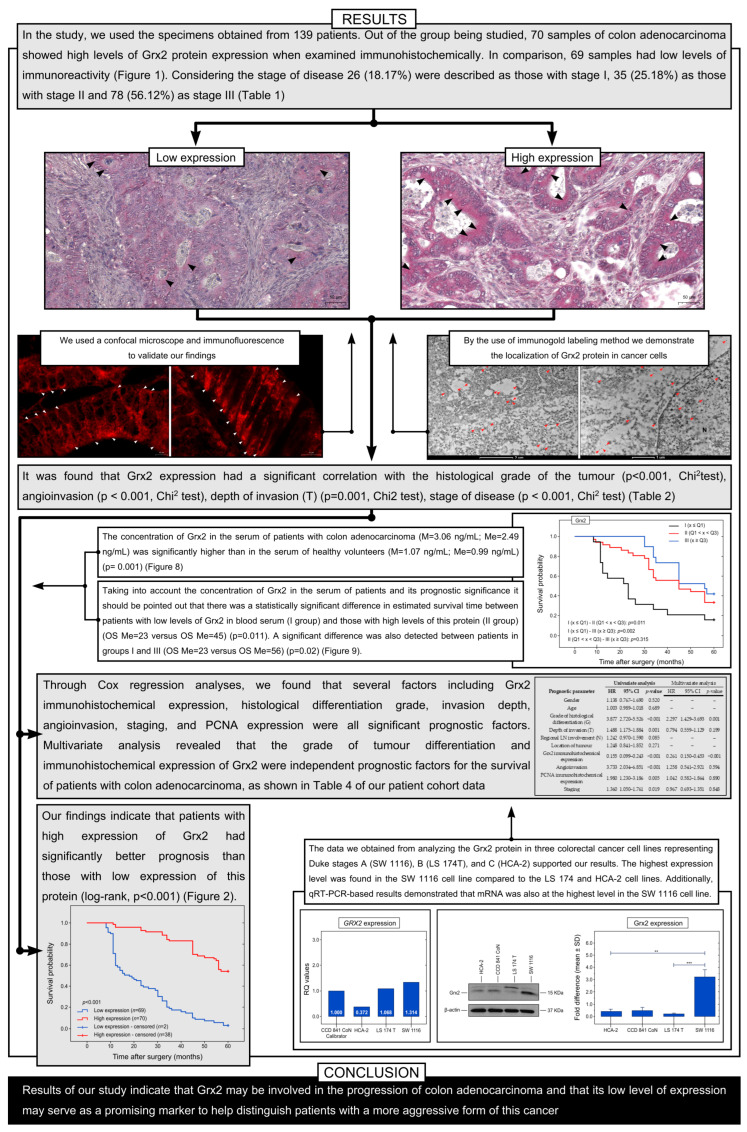
Schematic of the condensed presentation of our study results. ** *p*  <  0.01; *** *p*  <  0.001.

The schematic presentation of our results is shown in Figure 9.

## 3. Discussion

Grxs are enzymes that catalyze the deglutathionylation of protein-mixed disulfides with glutathione (protein-SSG), which can affect proteins regulating cellular processes. Any changes in their activity can interfere with apoptotic and survival signaling pathways, ultimately leading to pathological states by disrupting cell proliferation [15,17,21,27]. 

It is important to note that the *GRX2* gene and other oxidoreductases from the Trx family are often found to be upregulated in various types of carcinomas, leading to increased resistance to chemotherapy, increased proliferation of cancer cells and decreased survival rates of patients [31]. However, an inverse correlation has been reported in lung cancer patients, where a decreased level of Grx1 and Grx2 immunohistochemical expression was found [32]. In glioma, the expression of *GRX2* was decreased, but its transcription increased as the WHO grade of the tumors progressed [33]. In contrast, the high expression of *GRX2* was noted in patients with hepatocellular carcinoma and metabolic syndrome [34]. In clear cell renal carcinoma, the high expression of Grx2 protein in cancer tissue was associated with poor survival, and more locally advanced tumor stages [35]. 

Our research investigates the importance of Grx2 protein’s immunohistochemical expression as a potential biomarker in the diagnosis of adenocarcinoma, a type of colorectal cancer. Our study focuses on patients who have stage I, II, and III diseases, which includes patients with a primary tumor in the colon (I, II) and those with cancer metastases in the regional lymph nodes (III). We have excluded stage IV patients from our study as they are a complex and heterogeneous group requiring a special analysis. However, we plan to examine Grx2 protein’s expression in this group in the future. We have detected the immunohistochemical expression of Grx2 protein in both colon adenocarcinoma and non-pathological colon mucosa samples. We used a confocal microscope and immunofluorescence to validate our findings. Immunohistochemistry (IHC) is a technique used to assess the expression of biomarker proteins in tumor tissue. The staining’s appearance and intensity are visually and semiquantitatively evaluated. IHC is a simple and widely available technique that does not require expensive equipment. Combining conventional immunohistochemistry with immunofluorescence and digital image analysis (DIA) improves accuracy and provides controls for analyzing various proteins as potential biomarkers. Both techniques have confirmed the importance of Grx2 for prognosis, but DIA provides better visualization of the data and helps in identifying this antigen within the cells. It displays the red signals of varying intensities. The combination of immunohistochemistry and DIA is an effective and accurate method to measure protein expression in tissues. DIA’s fluorescence signal has a dynamic linear response that is much greater than chromogenic staining. This allows for a more precise and objective measurement of the amount of protein expression [36,37].

According to our research and statistical analysis, there is a strong connection between the expression of Grx2 protein and various factors that indicate cancer growth such as tumor histological grade (G), depth of invasion (T), staging, and angioinvasion. Our studies reveal that 93% of patients who have G3 differentiation grade have low levels of Grx2 expression. On the other hand, only 7% of patients who have G3 differentiation exhibit low levels of expression. As far as the depth of invasion is concerned, 92% of patients labeled T1 have high levels of Grx2 expression, whereas only 33% of patients in T4 have high levels of Grx2 expression. When angioinvasion is taken into account, approximately 87% of patients who do not have angioinvasion exhibit high levels of Grx2 expression. Further analysis of the expression of Grx2 protein in patients with colorectal adenocarcinoma revealed that 85% of patients with stage I, 31% of patients with stage II, and around 48% of patients with stage III exhibit high levels of Grx2 expression. These results are consistent with the data we obtained from analyzing Grx2 protein in three colorectal cancer cell lines, representing Duke stages A, B, and C. The highest expression level was found in the SW 1116 cell line compared to the LS 174 and HCA-2 cell lines. Additionally, qRT-PCR-based results demonstrated that mRNA was also at the highest level in the SW 1116 cell line. This suggests that Grx2 may be involved in the progression of colorectal cancer and that its low level of expression may serve as a promising marker to help distinguish patients with a more aggressive form of the tumor. In this context, it should be pointed out that Grx2 can regulate mitochondrial dynamics and function, and a low expression of this protein is associated with cardiac dysfunction in humans. It may also play a similar role in colon tissue [38,39]. Moreover, Grx2 absence impairs mitochondrial fusion, ultrastructure and energetics in primary cardiomyocytes and cardiac tissue. This is probably connected with an irreversible S-gluathionylation of mitochondrial proteins [40]. In lung cancer patients, the expression of Grx2 did not correlate with the expression of this protein in cancer cell lines. It has been found that the proliferation of lung cancer tissue, as determined by Mib-1, is inversely related to the expression of *GRX1* and *GRX2*. Interestingly, HeLa cells with high expression of *GRX2a*, the mitochondrial isoform, showed a significant increase in proliferation and resistance to apoptosis. The difference in proliferation between the tumor tissue and the Grx2a-overproducing cells was attributed to the cells lacking the mitochondrial localization signal domain, which made Grx2a cytosolic in these cells. It was observed that the proliferation rate of these cells was not affected when compared to the control cells, despite the difference in location [26,27]. 

The studies analyzing Grx2 levels in patients’ blood revealed that the serum highest levels were found in stage I patients, which was reflected in the survival curves. A higher level of Grx2 in the serum has been associated with a more favorable outcome. Biomarkers in the field of cancer detection can be various kinds of biochemical molecules that circulate in the blood and are often used in the diagnosis of colorectal cancer. They are detected via a quantitative assay of proteins in the blood or immunohistochemistry [41]. There are currently only two biomarkers available for monitoring patients with colorectal cancer (CRC). These biomarkers are carcinoembryonic antigen (CEA) and carbohydrate antigen 19-9 (CA 19-9) [41]. CEA is a high-molecular-weight glycoprotein that was first identified in 1965. It is found in embryonic tissue and colorectal cancer, and is used as a biomarker to monitor the progression of CRC. An increased level of CEA is a poor prognostic factor in resectable CRC and is correlated with the progression of the cancer [42]. The sensitivity of CEA (carcinoembryonic antigen) increases as the tumor stage rises. However, CEA levels decrease after tumor resection. It is important to note that high blood levels of CEA are not specific to colorectal cancer (CRC). They may also be associated with inflammatory bowel disease, liver disease, pancreatitis, or other malignancies. It is important to understand that only a small portion of CRC patients have elevated CEA levels at advanced stages, and therefore, CEA measurement is not an efficient method for CRC screening [43]. Compared to CEA, the CA19-9 antigen has lower sensitivity and specificity in detecting colorectal cancer. However, it can be used as a marker for identifying pancreaticobiliary tumors. CEA is still the preferred antigen for use as a prognostic marker after diagnosis and during follow-up [44]. Other biomarkers, such as tissue polypeptide-specific antigen (TPS) and tissue polypeptide antigen (TPA), detect fragments of cytokeratins 8, 18, and 19. However, they lack the required sensitivity and specificity for screening CRC [42]. It is worth mentioning that methylated SEPT9 DNA can be used as a blood marker for detecting colorectal cancer (CRC). In patients with early-stage CRC (stage 0–I), the detection range of this approach is between 57% and 64% [45]. When the detection of methylation of the SEPT9 promoter region in plasma is combined with FIT, the sensitivity is increased to 94% [46]. The positive rate of mSEPT9 was higher in individuals with advanced colorectal cancer (CRC) compared to those with early-stage CRC. Additionally, mSEPT9 was found to be more prevalent in CRC than in adenomas. The sensitivity of detection was not associated with the location of the tumor (whether on the left or right side), but it was found to be linked to the race of the subjects, with higher detection rates observed in Asian populations versus Caucasian populations [46]. 

By the use of immunogold labeling method, we demonstrate the localization of Grx2 protein in cancer cells. While the development of super-resolution microscopy at the level of light microscopy has allowed for the monitoring of the sub-cellular localization of molecules, electron microscopy (EM) provides higher-resolution imaging to match molecular localization at the ultrastructural level [47]. The immunogold labeling of intracellular proteins using antibodies at the EM level permits the localization of these proteins within whole cells [48]. Knowledge of the distribution and quantification of such proteins under various stimulation conditions may give significant insight regarding their functionality [48]. In our study, this method confirmed the cytoplasmic expression of Grx2 in the plasma membrane, endoplasmic reticulum membranes, and mitochondria. The Grx2 antigen was detected in the cytoplasm as well as some nuclei. This is not surprising as one of the variants of Grx2 is known to be expressed specifically in the nucleus [47,48]. We were interested in verifying the localization of Grx1 protein in tumor tissue, as this could be the basis for investigations related to the development of targeted cell therapy in the future. The different localization of the Grx2 protein may have different functions in cell biology, particularly in maintaining cellular redox homeostasis. Based on in vitro analysis, the effect of Grx2 in stably transfected cells appears to be dependent on subcellular location [27]. For instance, Zhang et al. have reported that the overexpression of Grx2 in HeLa cytosol has protected Trx1 from oxidative damage, indicating a supportive role of Grx2 in the cytosolic redox balance of cancer cells [49]. Li et al. found that mitochondrial Grx2 depletion exacerbated APAP-induced liver damage by impairing the thiol redox compensatory reaction and enhancing oxidative injury via the AIF signal pathway [50].

The GSH-based antioxidant redox system (GRS), the major regulator of cellular redox balance, may represent a novel therapeutic approach for overcoming the progression and chemoresistance of cancer cells [11]. Many cancer drugs are toxic and harmful to the body. Because of this, ways to make anti-cancer therapy more targeted and safe has been sought out. One way is using GSH, a substance found in high levels in tumors, to activate prodrugs. This process can lead to GSH depletion and the release of cancer-killing toxins [11]. Researchers have found that romidepsin, a drug used to treat cutaneous T-cell lymphoma and other peripheral T-cell lymphomas, can be activated by GSH. This activation occurs when GSH breaks a disulfide bond in the drug, creating a thiol group that binds to a zinc atom in a histone deacetylase [11]. Clinically, GSH itself is not effective and various progenitors or chemically altered analogs have been generated to mimic the different physiological or pharmacological responses of glutathione, for example, NAC (N-acetylcysteine; Mucomyst) and YM737, a monoester of GSH [51]. Another GSH analog approach is cysteine-substituted *S*-nitrosoglutathione. Since the identification of elevated levels of GST in resistant tumors, GSH analogs have been designed to differentially block GST isoforms. Telcyta (TLK-286) is an GSH analog that is used together with platinum, taxane, and anthracycline cytotoxic chemotherapies in a range of cancers that express very high levels of the glutathione S-transferase pi-1 gene (GST-P1-1) [52]. Telintra (TLK199) is a small-molecule GSTP-1 inhibitor being investigated as a potential preventive treatment for myelosuppression in blood diseases, namely myelodysplastic syndrome [53]. 

Studies have been conducted on drugs that target S-glutathionylation, which have shown direct anticancer effects. These drugs act on a range of signaling pathways. One of these drugs is identified as NOV-002. It has undergone a completed Phase III trial (NCT00347412) in advanced NSCLC and Phase II trials in breast and ovarian cancers. NOV-002 alters the GSH:GSSG ratio and induces S-glutathionylation. Research has shown that NOV-002-induced S-glutathionylation can inhibit the proliferation, survival, and invasion of myeloid cell lines. Additionally, NOV-002 has significantly improved the effectiveness of cyclophosphamide chemotherapy in a murine model of colon cancer [54,55]. Promising effects of NOV-002 have been observed in patients with stage IIIb/IV NSCLC when used in combination with standard chemotherapy in a randomised Phase II trial [56]. 

### Conclusions and Proposals for the Future Relating the Role of Grx2 in Colorectal Cancer

Our study demonstrates the immunohistochemical and immunofluorescence expression of Grx2 in colon adenocarcinoma tissue in patients from European populations at stages I, II, and III of the disease. Out of the group being studied, 70 samples of colon adenocarcinoma (which is 50.36%) showed high levels of Grx2 protein expression, whereas 69 samples (which is 49.64%) had low levels of immunoreactivity. The high expression of Grx2 is associated with a high survival rate in colorectal adenocarcinoma patients according to the Cox regression model. Multivariate analysis indicated that the histological differentiation grade and immunohistochemical expression of Grx2 in colon adenocarcinoma tissue might be regarded as independent prognostic factors. The Grx2 protein has been detected in the cytoplasm, mitochondria, cisterns of the rough endoplasmic reticulum, and nucleus of cancer cells. Studies analyzing Grx2 levels in patients’ blood confirmed that the highest levels of serum Grx2 protein were also found in stage I patients, which was reflected in the survival curves. These results were supported by in vitro analysis conducted on colorectal cancer cell lines that corresponded to stages I, II, and III of colorectal cancer, using qRT-PCR and Western blot. 

Nevertheless, our study has some limitations which need to be considered. First, the size of the cohort that we studied was limited, which may introduce a selection bias to the study. Therefore, future studies should be conducted to increase the sample size, and in vitro molecular experiments should be carried out to understand the mechanism of Grx2 activity. However, the advantage of our work is that we carefully selected a group of patients without any accompanying diseases. Our experiment is also a starting point for further clinical trials assessing the diagnostic utility of redox biomarkers in a larger population of colorectal cancer patients. Moreover, we suggest that some functional analyses in colorectal cancer cells, especially those obtained from patients, should be conducted to investigate how manipulation of Grx2 expression levels affects cellular behavior. The current study establishes correlations between Grx2 expression and various clinicopathological parameters, and provides a solid basis for the molecular studies that we are planning. These include: (A) the knockdown or overexpression of Grx2 in cells, and combination with drug treatment; (B) the relationship between PCNA and Grx2 (which is upregulated and which is downregulated) in cancer cells; and (C) investigating the functional significance of Grx2 in serum. This may provide insight into potential therapeutic interventions targeting Grx2. Furthermore, another future direction is to explore the development of drugs or interventions targeting Grx2 based on its role in cancer development, along with exploring the therapeutic potential of modulating Grx2 expression or activity.

## 4. Materials and Methods

### 4.1. Patients and Tumor Samples

For the present investigation, we obtained colon tissue specimens from people in whom colorectal adenocarcinoma was diagnosed following a histopathological diagnosis. The specimens were taken during a colon resection at Zabrze and Jaworzno Municipal Hospital between January 2014 and December 2018. We excluded subjects who had undergone preoperative radiotherapy or chemotherapy, had severe medical disorders or distant metastases, had inflammatory bowel disease, had a tumor recurrence, or had a different histopathological subtype than adenocarcinoma. We used a standard protocol to prepare tissue sections from all surgical samples, including fragments of tumor and surrounding tissue that were free of tumor changes. The specimens were fixed in formalin and embedded in paraffin blocks. The blocks were cut and sections routinely H&E-stained for histopathological diagnoses. We also examined margins, and if any cancer cells were detected, the material was excluded from analysis. To evaluate the prognostic importance of the Grx2 protein, we observed patients for 5 years (60 months).

### 4.2. Immunohistochemical Staining

Tissue blocks containing colon adenocarcinoma specimens and resected margins that were fixed in formalin were embedded in paraffin and then cut into 4 µm thick sections. These sections were then fixed on Polysine slides and deparaffinized using xylene and a graded series of alcohol. To retrieve the antigenicity, the tissue sections were treated with microwaves in a 10 mM citrate buffer (pH 6.0) for 8 min each. Following this, the sections were incubated with antibodies to Glutaredoxin2 (GeneTex, polyclonal antibody, Cat. No. GTX81538, final dilution 1:200, Irvine, CA, USA), which targeted the C-terminus epitope, and PCNA (GeneTex, polyclonal antibody, Cat. No. GTX100539, final dilution 1:600, Irvine, CA, USA). To visualize protein expression, the sections were treated with BrightVision (Cat. No. DPVB55HRP, WellMed BV, ’t Holland 31, 6921 GX Duiven, The Netherlands) and Permanent AP Red Chromogen (Dako LPR from Agilent Technologies Code K0640). For negative control, the primary antibody was omitted. Mayer’s hematoxylin was used to counterstain the nuclei. In order to analyze the results of immune histochemical staining, we followed the immunoreactive score used in previous publications [29,30,57,58]. The score was based on both the intensity and the number of cells with positive reactions. The intensity was graded as follows: 0 for no signal, 1 for weak, 2 for moderate, and 3 for strong staining. We assessed the frequency of positive cells semiquantitatively by evaluating the entire section, and each sample was scored on a scale of 0 to 4: 0 for negative, 1 for positive staining in 10–25% of cells, 2 for 26–50% of cells, 3 for 51–75% of cells, and 4 for 76–100% of cells. We then calculated the total score of 0–12 and graded it as follows: I for scores 0–1, II for scores 2–4, III for scores 5–8, and IV for scores 9–12. We considered grade I as negative, and grades II, III, and IV as positive. Grades I and II represented low expression (no or weak staining), and grades III and IV represented high expression (strong staining). The evaluation was carried out by two independent pathologists, and any differences in opinion were resolved by consensus.

### 4.3. Immunogold Electron Microscopy

Samples were fixed in a 4% solution of paraformaldehyde in 0.1M phosphate-buffered saline (PBS) at room temperature for two hours. The specimens were then rinsed several times in PBS. Next, the samples were dehydrated using a graded ethanol series and infiltrated for 30 min on ice in a 2:1 (*v*:*v*) ethanol/LR White mixture followed by a 1:2 (*v*:*v*) mixture. After that, the samples were infiltrated with pure LR White. Ultrasetions were immunolabeled and placed on Formvar-coated 200 mesh nickel grids. Sections on the grids were pre-incubated for 30 min by floating on drops of 50 mM NH4 Cl in PBS, followed by 30 min of blocking on drops of 1% BSA in PBS. To prepare the grids, a 1:20 dilution of primary anti-Grx2 antibody in BSA was applied and left overnight at 4 °C for 16–18 h. Following this, the sections were incubated with immunogold-conjugated goat anti-mouse IgG 15 nm (BBInternational BBI Solutions, Sittingbourne, UK) diluted in 1:100 for 1 h to detect the bound antibodies. The grids were then rinsed with PBS drops (five changes for 5 min each) and water (three changes for 3 min each) before staining with 0.5% aqueous uranyl acetate. Notably, the main antibody was not used in the negative controls. The grids were air-dried and subsequently examined at 120 kV in a TECNAI 12 G2 Spirit Bio Twin FEI Company transmission electron microscope. The resulting images were captured using a Morada CCD camera (Gatan RIO 9, Pleasanton, CA, USA).

### 4.4. Colorectal Cancer Cell Lines

For the experiments, three different colorectal cancer cell lines were used, HCA-2 (Duke C), LS 174T (Duke B), and SW 1116 (Duke A), along with a normal epithelial cell line known as CCD 841 CoN. SW 1116, LS 174T, and HCA-2 cell lines were provided by ATCC (American Type Culture Collection ATCC^®^, Old Town Manassas, VA, USA). The HCA-2 line was a donation from Prof. Magdalena Skonieczna of the Silesian University of Technology. The following cell lines were chosen to correspond as closely as possible to the different stages of cancer and reflect the cancer cells at each stage of the disease: the SW 1116 line corresponds to Duke A or stage I, the LS 174 line corresponds to Duke B or stage II, and the HCA-2 line corresponds to Duke C or stage III of the disease. Line SW 1116 (ref CCL233) was isolated from a Caucasian man aged 73. Line LS 174T (ref CL 188) is a cell with epithelial morphology that was isolated from the colon of a 58-year-old Caucasian woman with adenocarcinoma of the colon. This cell line was deposited by Northwestern University and can be used for cancer research. The line CCD 841 CoN line are the cells that have been isolated from normal human colon tissue. The cells resemble epithelial cells but do not contain keratin and there is no clear evidence of their epithelial origin.

The ideal cell growth conditions were ensured by using appropriate culture media. An Eagle’s minimum essential medium (EMEM) (ATCC 30-2003) was the choice of medium for the CCD 841 CoN and LS 174T cell lines, while Dulbecco’s modified Eagle’s medium/Nutrient Mixture F-12 Ham (DMEM) from Sigma-Aldrich D8437 was utilized for the SW 1116 and HCA-2 cell lines. To supplement both media, 10% fetal bovine serum (FBS) (ATCC 30-2020) and a 1% penicillin–streptomycin–neomycin stabilized solution (Sigma-Aldrich P4083) were added.

### 4.5. GRX2 Gene Expression in Colorectal Cancer Cell Lines

RNA was isolated using the RNA Isolation Kit (BioVendor, Brno, Czech Republic) according to the attached protocol. The qualitative and quantitative analyses of all isolated RNA was performed by spectrophotometry in a Biochrom WPA Biowave DNA UV/Vis Spectrophotometer (Biochrom, Business Park, Building 1020, 1010 Cambourne Rd, Cambourne, Cambridge CB23 6DW, UK). In the next stage of the investigation, complementary DNA–cDNA was synthesized from the isolated RNA via the reverse transcription reaction using a High-Capacity cDNA Reverse Transcription Kit with RNase Inhibitor (Applied Biosystems, ThermoFisher Scientific, Waltham, MA, USA) according to the manufacturer’s instructions. The relative gene expression analysis was performed employing a Quantitative Reverse-Transcription Polymerase Chain Reaction (qRT–PCR) using specific TaqMan^®^ Gene Expression Assays (Applied Biosystems, USA). Q-PCR was performed for the GLRX2 gene (Hs00375015_m1). The glyceraldehyde-3-phosphate dehydrogenase gene (GAPDH, Hs03929097_g1) was used as an endogenous control. The calibrator (reference sample) was a human CCD 841 CoN cell line. The qRT–PCR was performed in a volume of 20 µL using 1 µL of cDNA, 10 µL of TaqMan^®^ Gene Expression Master Mix (Applied Biosystems, USA), 1 µL of a primer and probe mix (TaqMan^®^ Gene Expression Assays), and 8 µL of H_2_O (EURx, Gdańsk, Poland). The thermal cycle for all analyzed genes was as follows: 95 °C for 10 min, followed by 40 cycles of 95 °C for 15 s, 60 °C for 1 min. All assays were conducted with a QuantStudio 5 RealTime PCR System (Applied Biosystems, USA). The comparative threshold cycle (Ct) method 2-∆∆Ct was used to determine the relative gene expression (RQ). The negative control included in a qPCR experiment was no template control (NTC) omits any cDNA template from a reaction, and serves as a general control for extraneous nucleic acid contamination.

For the Western Blot study, the cells were first washed with PBS and lysed in TLB. Next, 10 µg of protein was separated by SDS-PAGE (15%) for 1.5 h at 150 V, and transferred to a nitrocellulose membrane using wet blotting in a transfer buffer for 2 h at 300 mA and 4 °C. The membranes were treated with an Antigen Pretreatment Solution (SuperSignal Western Blot Enhancer, Thermo Scientific ThermoFisher Scientific, Waltham, MA, USA) for 10 min and then blocked using 5% skim milk in TBST for 30 min at room temperature. Primary antibody incubation was carried out overnight at 4 °C using Primary Antibody Diluent (46641; Thermo; SuperSignal Western Blot Enhancer). After washing with TBST, the membrane was incubated with HRP-conjugated secondary antibody in 5% skim milk/TBST for 1 h at room temperature. Detection was performed using SuperSignal© West Femto Maximum Sensitivity Substrate (34095; Thermo Scientific, Karlsruhe, Germany) and Amersham Hyperfilm ECL (28906839; GE Healthcare, Freiburg, Germany) films. The Western blotting process included the use of rabbit anti-Glutaredoxin 2 (GTX81538; 1:1000; GeneTex) and mouse anti-β-Actin (MAB8929; 1:1000; R&D SYSTEM), as well as HRP-coupled mouse anti-rabbit IgG (31464; 1:25,000; Invitrogen; Waltham, MA, USA) and goat anti-mouse IgG (A16078; 1:25,000; Invitrogen) antibodies, with the respective dilutions.

### 4.6. Serum Level of Grx2 in Colon Adenocarcinoma Patients

In this study, there were participants 74 patients with colon adenocarcinoma, consisting of 37 males and 37 females aged between 56 and 79 years. Additionally, there were 20 healthy volunteers who had no history of inflammatory diseases or cancers, acting as the control group. Blood samples were collected from the colon adenocarcinoma patients before treatment and stored at a temperature of −80 °C. The serum level of Grx2 was measured using the enzyme-linked immunosorbent assay (ELISA) kit SEG980Hu 96 Tests Enzyme-linked Immunosorbent Assay Kit for Glutaredoxin 2 (GLRX2) from Cloud-Clone Corp. Wuhan, China, following the manufacturer’s instructions. To analyze the data, all participants were divided into three groups based on the quartile range of Grx2 serum levels in the total group (refer to Table 5 for details).

### 4.7. Statistical Analysis

In this study, we analyzed the relationship between Grx2 immunohistochemical ex-pression and relevant clinical parameters using Statistica 9.1 software developed by StatSoft in Krakow, Poland. To evaluate all numerical variables, we used the statistical measures of median and range. We assessed the relative characteristics of the groups studied using the chi-squared test (Ch^2^ test). In the case of the correlation between Grx2 and PCNA, treated as dependent variables, we used the McNemara test. The study assessed the relationship between Grx2 expression and patient survival using Kaplan–Meier analysis and log-rank tests. Statistical significance was set at *p* < 0.05. A similar test was used to assess the probability of colon adenocarcinoma patients in relation to Grx2 serum level. For the analysis of the relationship between clinical parameters and Grx2 serum level, the Wilcoxon paired *t*-test was used.

## Figures and Tables

**Table 1:** Characteristics of the patients included in the study (n = 139).

		N (Number of Cases)	%
Gender	Females	70	50.36
	Males	69	49.64
Age [years]	≤60 years	56	40.29
	61–75 years	45	32.37
	>75 years	38	27.34
	M ± SD	63.87 ± 13.29	
	Me [Q1–Q3]	64 [age range: 54–77]	
	Min–Max	33–89	
Grade of histological differentiation (G)	G1	26	18.70
	G2	72	51.80
	G3	41	29.50
Depth of invasion (T)	T1	23	16.55
	T2	17	12.23
	T3	78	56.11
	T4	21	15.11
Regional lymph node involvement (N)	N0	58	41.73
	N1	46	33.09
	N2	35	25.18
Location of tumor		75	53.96
	Distal	64	46.04
Angioinvasion	No	32	23.02
	Yes	107	76.98
PCNA Immunohistochemical expression	Low	42	30.22
	High	97	69.78
Staging	I	26	18.70
	II	35	25.18
	III	78	56.12

**Table 2:** Associations between the expression of Grx2 protein (dependent variable) and clinical characteristics in colon adenocarcinoma patients (independent variables).

		The Immunoexpression Level of Grx2				*p*-Value
		Low		High		
Age [Years]	≤60 years	28	(50.00%)	28	(50.00%)	Chi2 = 0.614 df = 2 *p* = 0.736
	61–75 years	24	(53.33%)	21	(46.67%)	
	>75 years	17	(44.74%)	21	(55.26%)	
Gender	Females	35	(50.00%)	35	(50.00%)	Chi2 = 0.007 df = 1 *p* = 0.932
	Males	34	(49.28%)	35	(50.72%)	
Grade of histological differentiation (G)	G1	2	(7.69%)	24	(92.31%)	Chi2 = 15.426 df = 3 *p* < 0.001
	G2	29	(40.28%)	43	(59.72%)	
	G3	38	(92.68%)	3	(7.32%)	
Depth of invasion (T)	T1	4	(17.39%)	19	(82.61%)	Chi2 = 15.426 df = 3 *p* = 0.001
	T2	6	(35.29%)	11	(64.71%)	
	T3	45	(57.69%)	33	(42.31%)	
	T4	14	(66.67%)	7	(33.33%)	
Regional lymph node involvement (N)	N0	27	(46.55%)	31	(53.45%)	Chi2 = 3.366 df = 2 *p* = 0.186
	N1	20	(43.48%)	26	(56.52%)	
	N2	22	(62.86%)	13	(37.14%)	
Location of tumor	Proximal	35	(46.67%)	40	(53.33%)	Chi2 = 0.576 df = 1 *p* = 0.448
	Distal	34	(53.13%)	30	(46.88%)	
Angioinvasion	No	4	(12.50%)	28	(87.50%)	Chi2 = 22.938 df = 1 *p* < 0.001
	Yes	65	(60.75%)	42	(39.25%)	
PCNA Immunohistochemical expression	Low	16	(38.10%)	26	(61.90%)	Chi2 = 3.209 df = 1 *p* = 0.073
	High	53	(54.64%)	44	(45.36%)	
Staging	I	4	(15.38%)	22	(84.62%)	Chi2 = 17.489 df = 2 *p* < 0.001
	II	24	(68.57%)	11	(31.43%)	
	III	41	(52.56%)	37	(47.44%)	

**Table 3:** Correlations between the expression of Grx2 protein and PCNA protein (as dependent variables).

		The Immunoexpression Level of Grx2				*p*-Value
		Low		High		
PCNA expression	Low	16	(11.51%)	26	(18.71%)	*p* < 0.001
	High	53	(38.13%)	44	(31.65%)	*p* = 0.003

**Table 4:** Univariate and multivariate evaluations of selected clinical parameters in patients with colon adenocarcinoma using Cox regression analysis.

Prognostic Parameter	Univariate Analysis			Multivariate Analysis		
	HR	95% CI	*p*-Value	HR	95% CI	*p*-Value
Gender	1.138	0.767–1.690	0.520	–	–	–
Age	1.003	0.989–1.018	0.689	–	–	–
Grade of histological differentiation (G)	3.877	2.720–5.526	<0.001	2.297	1.429–3.693	0.001
Depth of invasion (T)	1.488	1.175–1.884	0.001	0.794	0.559–1.129	0.199
Regional LN involvement (N)	1.242	0.970–1.590	0.085	–	–	–
Location of tumour	1.248	0.841–1.852	0.271	–	–	–
Grx2 immunohistochemical expression	0.155	0.099–0.243	<0.001	0.261	0.150–0.453	<0.001
Angioinvasion	3.733	2.034–6.851	<0.001	1.258	0.541–2.921	0.594
PCNA immunohistochemical expression	1.980	1.230–3.186	0.005	1.042	0.582–1.864	0.890
Staging	1.360	1.050–1.761	0.019	0.967	0.693–1.351	0.848

**Table 5:** Grx2 serum levels of colon adenocarcinoma patients.

	Grx2 Serum Levels of Colon Adenocarcinoma Patients								
	N	%	M	Me	Min	Max	Q1	Q3	SD
x ≤ Q1	19	25.68	0.48	0.39	0.01	1.00	0.28	0.79	0.28
Q1 < x < Q3	36	48.65	2.59	2.49	1.21	4.50	2.05	3.23	0.95
x ≥ Q3	19	25.68	6.52	6.35	4.66	9.11	5.01	7.88	1.48

## Data Availability

Data are contained within the article.
